# Hybrid CNN-GNN architectures with distributed training for heathland plant classification

**DOI:** 10.1007/s10661-026-15190-8

**Published:** 2026-06-06

**Authors:** Babatoundé Moctard Olouladé, Jesper Leth Bak, Peter Borgen Sørensen, Haomin Yu, Christian Damgaard

**Affiliations:** 1 Department of Ecoscience, Aarhus University, C.F. Møllers Alle, Aarhus, 8000 Denmark; 2 School of Sciences, Engineering and Environment, University of Salford, 43 Crescent, Salford, M5 4WT UK

**Keywords:** Plant species classification, Land use, Ecological monitoring, Deep neural networks

## Abstract

Accurate predictions of heathland plant species are crucial for ecological monitoring and assessing biodiversity. Previous research has predominantly utilised convolutional neural networks (CNNs), which process images arranged on a regular grid. Although CNNs are effective at extracting local visual features, they frequently do not capture the irregular spatial relationships characteristic of heathland vegetation. Earlier approaches employing graph neural networks (GNNs) for plant classification have generally relied on simple or dataset-wide graph constructions, with limited consideration for edges that represent the actual morphological structure of plants. This study presents PlantGraphNet, a hybrid CNN–GNN framework that integrates visual and structural information for heathland plant species classification. PlantGraphNet extracts image-specific keypoints and local descriptors to construct graphs, where nodes correspond to plant regions and edges encode spatial relationships. The resulting graphs are processed using graph convolution and graph attention layers to capture relational context, while a CNN backbone provides complementary appearance features. These two modalities are combined into a unified representation for classification. To ensure scalability, PlantGraphNet employs distributed data-parallel training, enabling efficient gradient synchronisation across devices and near-linear performance scaling. When evaluated on Danish aerial heathland datasets, PlantGraphNet achieves a precision of 98.98%, substantially outperforming CNN-only baselines. In addition to improved accuracy, the explicit graph construction increases interpretability by associating classification outcomes with specific plant structures. These findings indicate that integrating CNN-derived features with purposefully designed graph representations of plant morphology yields a robust and interpretable approach for fine-grained heathland plant species classification.

## Introduction

Heathlands are semi-natural ecosystems with low plant species diversity, dominated by dwarf shrubs, and typically found on nutrient-poor sandy soils. These ecosystems perform essential functions, including carbon sequestration in biomass and soils and regulation of hydrological cycles (Field et al., 2017). Vegetation in heathlands reduces soil erosion and mitigates flood risk (Acreman & Holden, 2013). Anthropogenic pressures such as urbanisation, elevated nitrogen deposition, intensified agriculture, and climate change compromise the ecological integrity of heathlands. These disturbances facilitate tree encroachment, diminish native biodiversity, and reduce the ecosystem services provided by heathlands (Damgaard, 2025a; Stanton et al., 2025). Conservation and restoration strategies are required to sustain the long-term functionality and resilience of these ecosystems (Agboola et al., 2022; Aszalós et al., 2022; Damgaard, 2025b; Damgaard & Irvine, 2019; Rittl et al., 2025). Accurate mapping and taxonomic identification of heathland vegetation are critical for effective monitoring and evidence-based management. Current plant abundance assessments use plot-based approaches, in which species cover is manually estimated within small sample plots (Damgaard & Irvine, 2019; Kollert et al., 2021). These approaches are labour-intensive, require specialised expertise, and are limited in spatial coverage. Additionally, the subjectivity of visual assessments introduces variability and reduces reproducibility across surveys and time periods.

With the increasing availability of high-resolution imagery from field-deployed cameras, unmanned aerial systems, and mobile devices, there is a growing need for automated, scalable, and interpretable systems capable of performing fine-grained plant classification under diverse and often challenging conditions. Various studies have explored the use of deep learning algorithms, particularly convolutional neural networks (CNNs), to enhance the accuracy and efficiency of plant species classification (Cai et al., 2023; Chen et al., 2018; Dyrmann et al., 2016). Models like ResNet50 (He et al., 2016), AlexNet (Krizhevsky et al., 2017), MobileNetV2 (Dong et al., 2020), and GoogleNet (Szegedy et al., 2015) have achieved strong results by learning hierarchical visual characteristics from images of leaves or canopies of plants (Barbedo, 2019; Dyrmann et al., 2016; Espitalier et al., 2025; Ge et al., 2025). (Wagner et al., 2020, 2019) applied U-Net (Ronneberger et al., 2015) to establish the efficacy of the convolutional network in effectively delineating the types and disturbances of heathland within the Atlantic rainforest, using high-resolution images. Their results revealed that the U-net model could precisely segment natural forests and eucalyptus plantations, achieving a high degree of precision. Similarly, Guo et al. (2020, 2021) introduced an extensive deep-fusion model designed to leverage high-resolution satellite imagery for detailed forest mapping at the level of individual tree species. Their research underscored the importance of multitemporal data in achieving high classification accuracy, with reported results reaching 94.8 % when using Sentinel-2 imagery. However, the investigation by Cho et al. (2015) highlighted the challenges inherent in mapping rare or nondominant over-storey plant species, especially within the complex structure of heathland ecosystems. Their findings suggest that although high-resolution imagery can aid in identifying dominant species, precisely mapping less prevalent species remains challenging due to their frequent, indistinct presence within the canopy. Hościło and Lewandowska (2019) reiterates this limitation, indicating that although their methodologies attained high precision in forest type classification, the complexities inherent in species-level differentiation within heathlands necessitate further refinement of the employed algorithms. Integrating semi-supervised and weakly supervised learning techniques has emerged as a promising avenue for addressing some of these limitations. Vermeer et al. (2023) utilised CNN models to explore the potential of these approaches to enhance plant species classification.

Convolutional neural networks (CNNs) have proven effective in computer vision tasks, but they rely on rigid, grid-like data. The reliance on grid data may limit their performance in classifying plant species whose structural morphology is taxonomically significant. In fact, in plant classification, fine-grained morphological cues, such as leaf venation patterns, stem-leaf arrangements, and interleaf distances, are often critical in distinguishing between morphologically similar species. Standard CNNs are prone to overemphasising superficial features such as colour and texture, which are susceptible to variation caused by lighting, occlusion, and viewpoint changes. Moreover, the computational resources required for training deep learning models, especially when dealing with large datasets, make this task more challenging for scientists with constrained resources. This issue is especially pertinent in remote sensing applications, where high-resolution images can produce large amounts of data that require efficient processing (Bueso-Bello et al., 2023).

Graph neural networks (GNNs) effectively handle non-Euclidean data and are applied in many fields, including but not limited to computer vision (Diao et al., 2024; Han et al., 2022; Zhang et al., 2021), plant disease detection (Maruthai et al., 2025; Reddy et al., 2025) environmental monitoring (Baskar et al., 2025; Verma et al., 2023), and urban planning (Mostafazadeh et al., 2024; Zheng et al., 2023). GNNs effectively capture the relational dynamics that CNNs may not recognise (Oloulade et al., 2021; Scarselli et al., 2008). However, GNNs have not been extensively used in classifying heathland plant species despite the intricate morphological structures present in plant species. Existing approaches employing graph neural networks (GNNs) for plant classification have generally relied on basic or dataset-wide graph constructions, with limited consideration for edges that represent the actual morphological structure of plants (Maruthai et al., 2025; Mulugeta et al., 2024; Reddy et al., 2025; Zhong et al., 2024).

Accurate classification of heathland plant species is not only a technical objective but also a prerequisite for effective ecological management. In operational monitoring workflows, even small classification errors can propagate into biased estimates of species abundance, misidentification of invasive or encroaching species, and suboptimal land management decisions. Consequently, incremental improvements in classification accuracy, even on the order of one percentage point, can correspond to meaningful ecological impact when applied at the landscape scale, where millions of observations may be aggregated to inform conservation and restoration strategies. From this perspective, improving robustness and consistency across morphologically similar vegetation classes is particularly critical.

We propose PlantGraphNet, a novel graph-based framework for the classification of plant species that captures both structural and contextual morphological cues in a modular and extensible manner, including: (1) a hybrid GNN-CNN architecture that fuses graph-level relational reasoning with global visual feature extraction and (2) an implementation of a distributed training pipeline to handle high-resolution inputs efficiently and accelerate training. In this paper, the model is applied and tested in heathland classification, demonstrating superior performance compared to several baseline CNN architectures in terms of accuracy and robustness. Our architecture combines CNN-derived global descriptors with localised relational reasoning from GNNs, capturing interregional dependencies and subtle morphological patterns. To improve scalability and efficiency in handling large, high-resolution plant images, we utilise the distributed data-parallel (DDP) training paradigm, facilitating memory-efficient training and faster convergence across multiple computational nodes. Our computational analysis shows that the hybrid model achieves superior performance in fine-grained plant species benchmarks, outperforming established CNN baselines such as VGG16 (Liu & Deng, 2015), VGG19 (Simonyan & Zisserman, 2019), ResNet50 (He et al., 2016), ResNet101 (He et al., 2015), AlexNet (Krizhevsky et al., 2017), MobileNetV2 (Dong et al., 2020), GoogleNet (Szegedy et al., 2015), and YOLOv8 (Vaghela et al., 2025).

The key contributions of this work are as follows.

- We propose PlantGraphNet, a novel scalable hybrid GNN-CNN architecture that effectively integrates graph-level relational reasoning with global visual feature extraction.
- We design a feature fusion mechanism that seamlessly combines structural information from graphs with visual cues from convolutional networks, enhancing model expressiveness.
- We demonstrate that PlantGraphNet achieves state-of-the-art accuracy and robustness on heathland plant species classification, surpassing several standard CNN architectures.

## Materials and methods

### Dataset

#### Study site and data acquisition

**Fig. 1:**
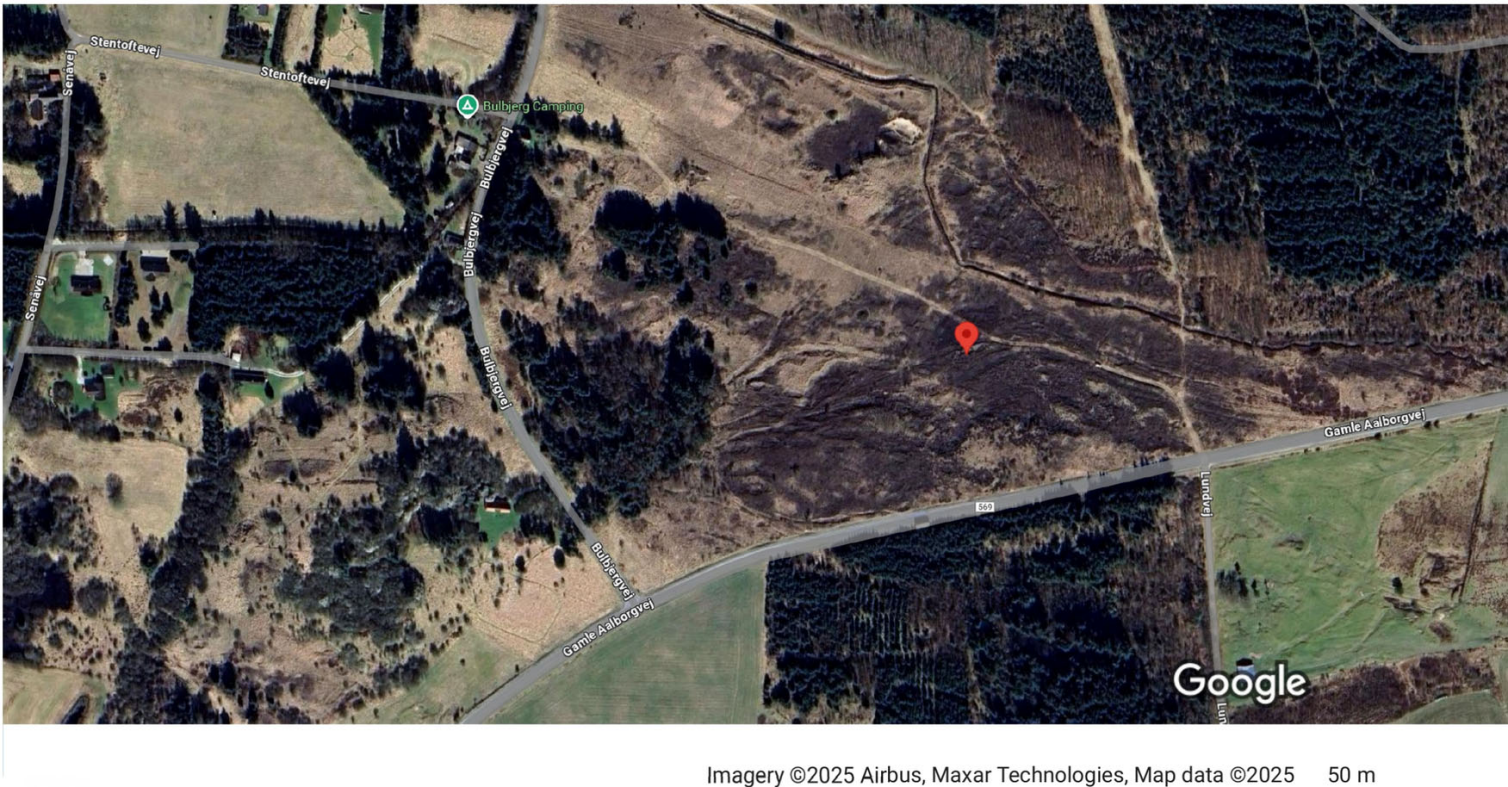
Experimental area at Vust Heath in Northern Jutland, Denmark

The dataset was collected from an experimental 12-hectare dune heath area located at Vust Heath^1^ in Northern Jutland, Denmark ($57.12317^{\circ }\text {N}$, $9.01179^{\circ }\text {E}$; see Fig. 1). This site is part of NATURA 2000 area no. 16, *Løgstør Bredning, Vejlerne og Bulbjerg*. Historically, Vust Heath formed part of a larger coastal heathland, but large-scale afforestation with spruce and pine during the 19th and 20th centuries significantly reduced its size.

**Fig. 2:**
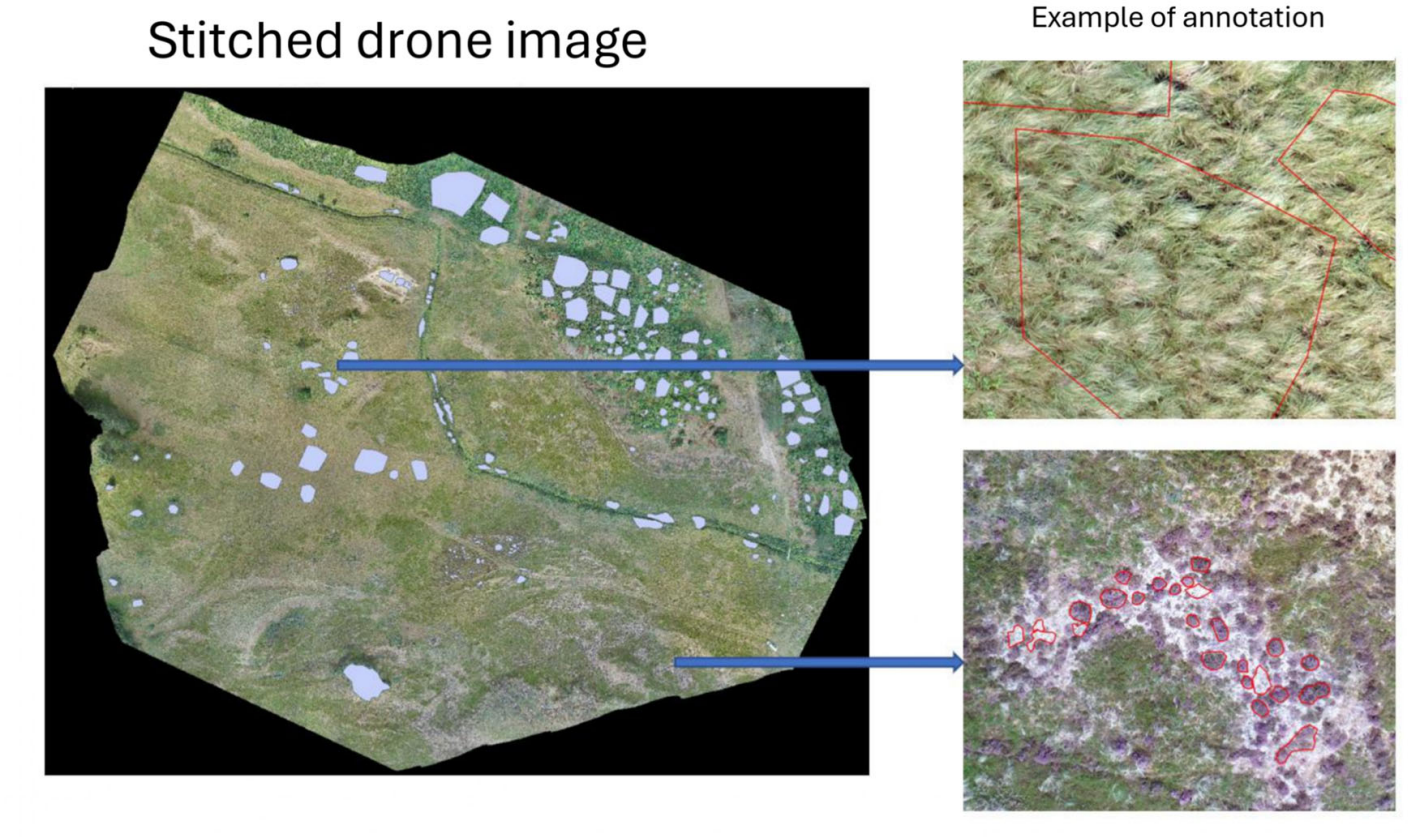
Stitched orthomosaic generated using ArcSoft with annotation

Aerial imagery was captured using a drone-mounted RGB camera under stable lighting conditions to ensure radiometric consistency and provide high-resolution detail. The captured images were subsequently processed with ArcSoft, a photogrammetry and image stitching software, to produce a high-resolution orthomosaic. The resultant stitched image has dimensions of 37,155 $\times$ 32,783 pixels, with a ground sampling distance (GSD) of 1.2 cm per pixel (Fig. 2). The image stitching process was carried out for the following purposes: (i) to assign precise geospatial coordinates to each pixel for accurate ground-truth alignment, (ii) to integrate visual data from overlapping images, and (iii) to enable efficient annotation of continuous vegetation structures.

#### Preprocessing and data split

**Fig. 3:**
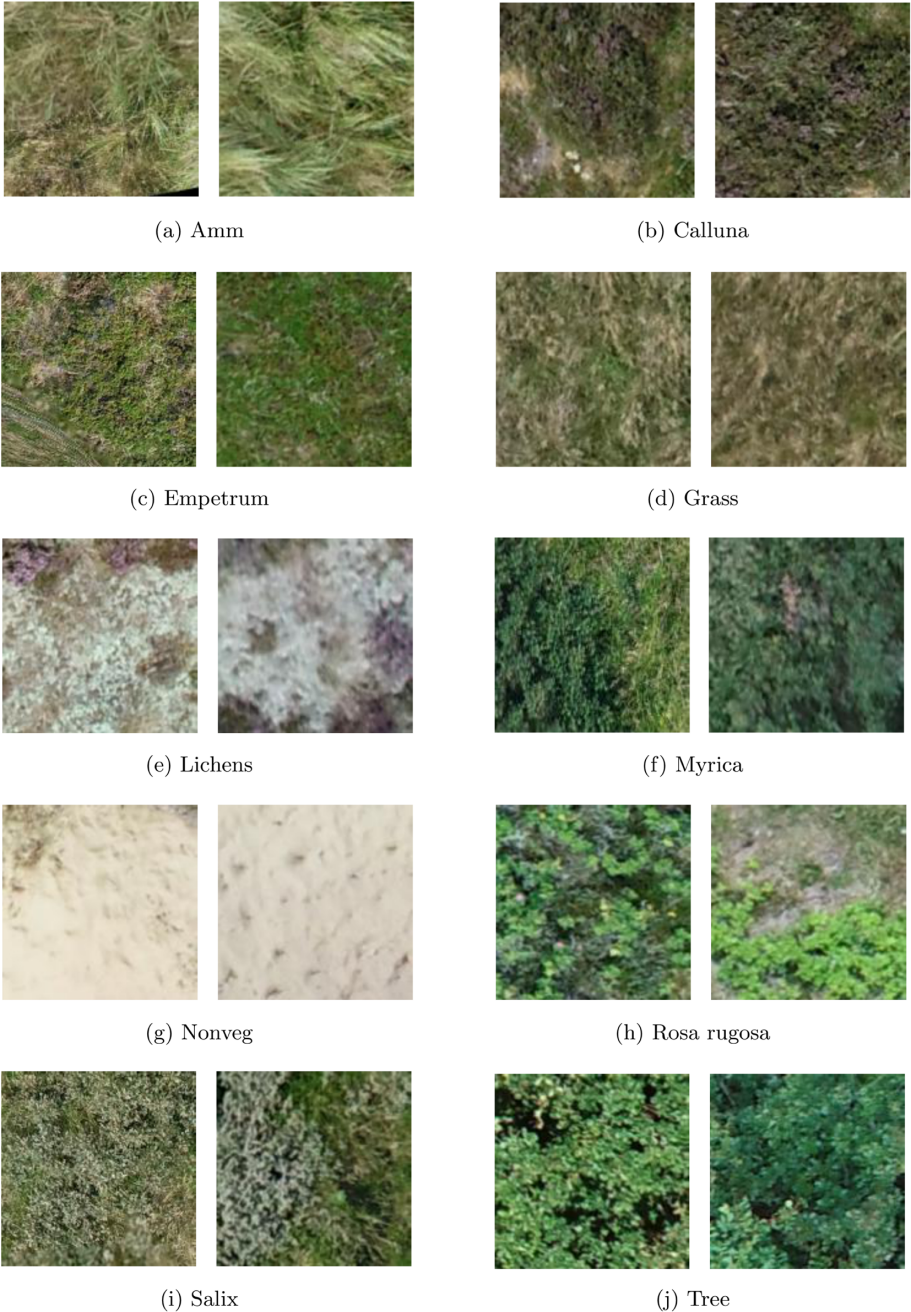
Representative image samples for each class in the dataset, including various vegetation types and non-vegetated areas. These samples illustrate the diversity and complexity of the categories used for model training and evaluation

We conducted manual annotation of the orthomosaic image. Manual annotation was performed by trained ecologists using GIS software. Polygons were delineated and classified into one of 10 predefined land cover classes, comprising 10 vegetation types, bare soil, and nine species or species groups (e.g., trees, lichens). These annotations correspond to ecologically significant boundaries and were validated by domain experts to ensure the accuracy of the labelling. The annotated polygons were extracted as images of $150 \times 150$ pixels. Figure 3 shows examples of the three vegetation types. This patch size was selected to strike a balance between ecological resolution and computational feasibility. Standard image augmentation techniques were applied to augment training data and enhance model robustness, including random rotations, horizontal and vertical flipping, and zooming. Our dataset consists of 12,140 images sourced from the annotated orthomosaic. We split the dataset into training, validation, and test sets, using 60% (7281 images) for training, 20% (2424 images) for validation, and 20% (2435 images) for testing.

### Methods

The proposed method constructs graph representations of plant images by detecting SIFT keypoints and connecting them using k-nearest neighbour relationships, which preserves spatial structure for relational learning. These graph-based features are combined with image-based features within a hybrid GNN-CNN architecture, as illustrated in Fig. 5, to facilitate multimodal classification. Model training is performed efficiently using a distributed data-parallel framework.

**Algorithm 1:**
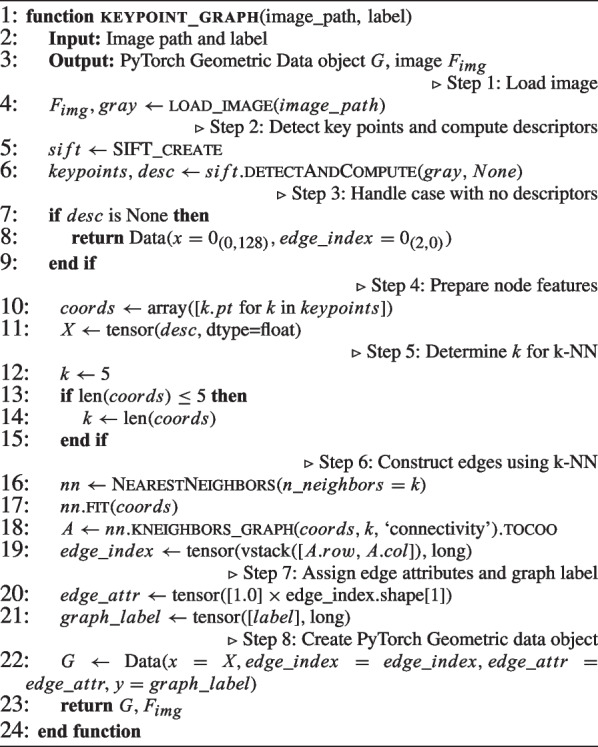
Keypoint-based graph construction method

#### Graph dataset construction from images

**Fig. 4:**
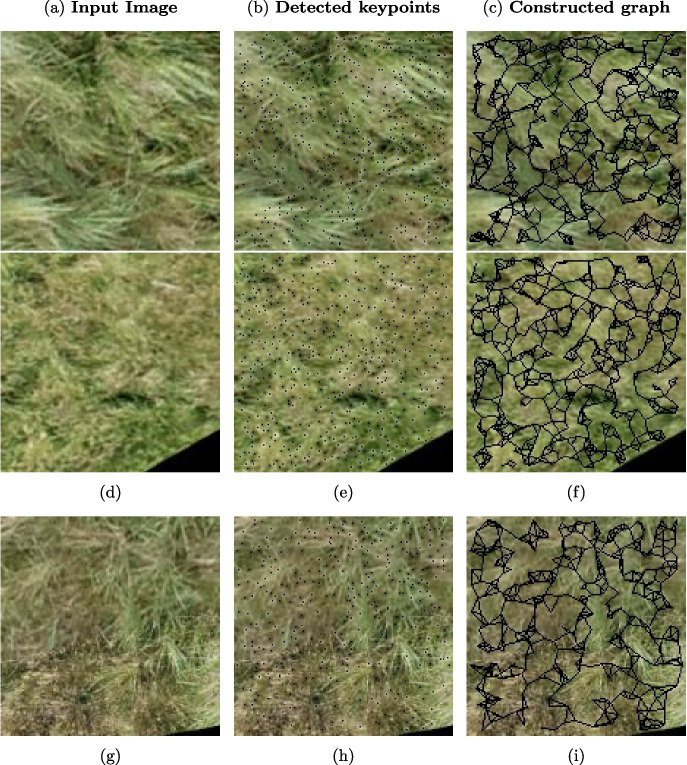
Sample images of constructed graphs

Graph-based representations of plant images are used to preserve the spatial relationships between local features, which are essential for robust analysis (Algorithm 1). Graph representations encapsulate the spatial structure among image regions, enabling learning algorithms to incorporate spatial context effectively. Specifically, we adopt keypoint-based graphs, where each keypoint refers to a distinctive location in the image (e.g., corners or blobs) robust to scale and rotation. These key points are detected using the Scale-Invariant Feature Transform (SIFT) algorithm (Jihad et al., 2025; Low, 2004). Each detected keypoint is treated as a node in the graph and is associated with a 128-dimensional feature vector $\textbf{x}_i \in \mathbb {R}^{128}$, corresponding to its SIFT descriptor. Edges are formed by connecting each node to its k-nearest neighbours in the 2D spatial domain, where pairwise distances are computed using the Euclidean metric. The value of k is set to 5 when at least five key points are detected; otherwise, it is adjusted to match the total number of available key points. The resulting edge connections define the graph’s edge index structure, and each edge attribute $e_{ij}$ is assigned a constant value of 1.0. Figure 4 illustrates the original input image, the detected key points, and the graph that has been constructed from these key points.

**Fig. 5:**
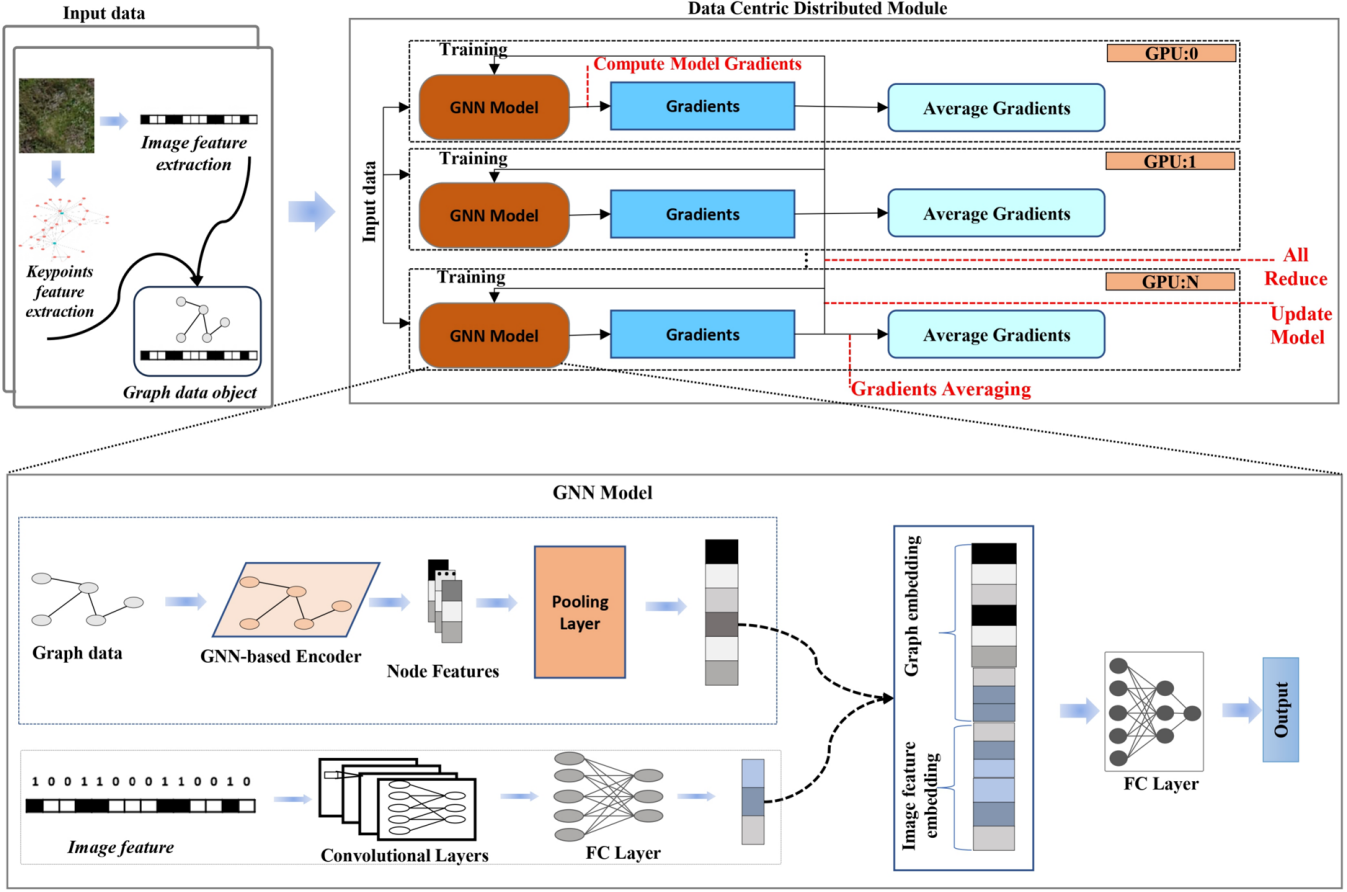
Overview of the proposed framework. GraphPlantNet processes plant images by converting them into graph data via key points and features. A graph neural network encodes these graphs, and features are fused with image-based features and then refined through neural layers to produce predictions. Training is efficiently distributed across multiple GPUs using a data-centric strategy and all-reduce for gradient updates

#### Hybrid GNN-CNN architecture

We propose a hybrid neural architecture that synergistically integrates graph-structured data and image-based information for improved multimodal classification. The model incorporates a GNN backbone for relational reasoning over structured inputs alongside a CNN module for extracting high-level semantic representations from images. This architectural design enables joint modelling of topological and spatial contexts (Fig. 5).

##### Graph representation learning

Let $G = (V, E)$ be a graph, where *V* denotes the set of nodes and *E* is the set of edges. In GNNs, a neural “layer” is reformulated as a two-step message-passing framework. At each layer, *k*, the representation of a node $i \in V$ is updated by first aggregating information from its neighbourhood $\mathcal {N}(i)$ using an aggregation function $\textsc {Agg}(\cdot )$ to compute $h^k_{\mathcal {N}(i)}$. This aggregated information is then combined with the previous representation $h^{k-1}_i$ of the node using a combination function $\textsc {Comb}(\cdot )$. The result is processed through a series of transformations: normalisation $\textsc {Norm}(\cdot )$, which stabilises training by reducing internal covariate shift; dropout $\partial (\cdot )$, which serves as a regularisation technique to prevent overfitting by randomly zeroing some nodes; and a non-linear activation function $\sigma (\cdot )$, which introduces non-linearity into the model (Cai et al., 2021). The update procedure is formally expressed as:

1 $$\begin{aligned} h^k_{\mathcal {N}(i)}= &  \textsc {Agg} \left( \left\{ h^{k-1}_j \mid j \in \mathcal {N}(i) \right\} \right) , \end{aligned}$$

2 $$\begin{aligned} h^k_i= &  \sigma \left( \partial \left( \textsc {Norm} \left( \textsc {Comb}(h^{k-1}_i, h^k_{\mathcal {N}(i)}) \right) \right) \right) , \end{aligned}$$

where $h^0_i = x_i$ represents the initial feature vector of node *i*, and $\partial (\cdot )$ denotes the dropout operator.

Our GNN model consists of three graph convolutional layers, $g^{(1)}, g^{(2)}, g^{(3)}$, applied over a batch of *B* graphs. To accommodate graphs with a variable number of nodes, all node features are concatenated into a single tensor $\textbf{X} \in \mathbb {R}^{N_{\text {total}} \times F}$, where $N_{\text {total}}$ is the total number of nodes across the batch, and *F* is the feature dimension. Edge features $\textbf{E}$, edge indices $\textbf{A}$, and a batch vector $\textbf{B} \in \mathbb {N}^{N_{\text {total}}}$, which maps each node to its graph, are also included as inputs.

Each convolutional layer follows a unified structure comprising graph convolution, normalisation, Dropout, and activation. The first layer is expressed as:

3 $$\begin{aligned} \textbf{H}^{(1)} = \sigma \left( \partial \left( \textsc {Norm}_1 \left( g^{(1)}(\textbf{X}, \textbf{E}, \textbf{A}, \textbf{B}) \right) \right) \right) , \end{aligned}$$

with subsequent layers $l \in \{2, 3\}$ defined similarly:

4 $$\begin{aligned} \textbf{H}^{(l)} = \sigma \left( \partial \left( \textsc {Norm}_l \left( g^{(l)}(\textbf{H}^{(l-1)}, \textbf{E}, \textbf{A}, \textbf{B}) \right) \right) \right) . \end{aligned}$$

To derive a graph-level embedding, global additive pooling is performed over the node embeddings of the final layer:

5 $$\begin{aligned} \textbf{h}_{\text {graph}} = \textsc {GlobalAddPool}(\textbf{H}^{(3)}, \textbf{B}), \end{aligned}$$

yielding $\textbf{h}_{\text {graph}} \in \mathbb {R}^{B \times d}$, where *d* is the output dimension. This pooled representation is then passed through a fully connected layer:

6 $$\begin{aligned} \textbf{z}_{\text {graph}} = \textbf{W}_g \textbf{h}_{\text {graph}} + \textbf{b}_g, \end{aligned}$$

where $\textbf{W}_g \in \mathbb {R}^{d \times d}$ is initialised using Xavier uniform initialisation and $\textbf{b}_g \in \mathbb {R}^d$ is a zero-initialised bias term.

##### Image representation learning

To efficiently extract semantically significant features from raw plant imagery, we leverage the capabilities provided by *pretrained CNNs*, which function as robust visual encoders due to their extensive training on large-scale annotated datasets. Specifically, the ResNet-50 architecture, pre-trained on ImageNet-1K, a benchmark database encompassing more than one million natural images, is utilised. This pretraining enables the network to acquire hierarchical visual representations, ranging from low-level edges to complex semantic structures, which effectively generalise to domain-specific imagery, such as photographs of plants. Given a set of input images $\textrm{I} \in \mathbb {R}^{B \times 3 \times H \times W}$, the ResNet-50 encoder processes each image to produce a global feature vector through the global average pooling of the final convolutional block. This methodology produces a feature tensor $\textrm{F}_{\text {img}} \in \mathbb {R}^{B \times d_{\text {img}}}$, where $d_{\text {img}}$ denotes the dimension of the final layer in ResNet-50.

We apply a linear transformation to map these features into a *shared embedding space* (e.g., for multimodal learning or downstream tasks).

$$ \textrm{z}_{\text {img}} = \textrm{W}_i \textrm{F}_{\text {img}} + \textrm{b}_i $$

where $\textrm{W}_i \in \mathbb {R}^{d \times d_{\text {img}}}$ is a learnable projection matrix and $\textrm{b}_i \in \mathbb {R}^d$ is a bias term, initialised to zero. The resulting embeddings $\textrm{z}_{\text {img}} \in \mathbb {R}^{B\times d}$ provide compact and transferable representations of the input images.

##### Multimodal fusion and classification

In the multimodal configuration, the final joint representation $\textrm{z}$ is constructed by concatenating the learned embeddings from the graph and image modalities, followed by dropout regularisation:

$$ \textrm{z} = \text {Dropout} \left( \left[ \textrm{z}_{\text {graph}} \, \Vert \, \textrm{z}_{\text {img}} \right] \right) $$

Here, **Dropout** is used as a stochastic regularisation technique that randomly zeroes a proportion *p* of the input units during training. It mitigates the risk of overfitting by discouraging the co-adaptation of features. This work uses a dropout rate of $p = 0.2$.

The resulting multimodal representation $\textrm{z}$ is then passed through a linear classifier to compute the class logits:

$$ \textrm{y} = \textrm{W}_c \textrm{z} + \textrm{b}_c $$

where $\textrm{W}_c \in \mathbb {R}^{C \times 2d}$ is the learned weight matrix, $\textrm{b}_c \in \mathbb {R}^{C}$ is the bias term, and *C* denotes the number of target classes.

The classification pipeline is adapted accordingly in the **unimodal** setting, where only a single modality is utilised. For instance, in the graph-only variant, the image embedding $\textrm{z}_{\text {img}}$ is omitted, and classification is performed directly on the graph representation:

$$ \textrm{y} = \textrm{W}_c' \textrm{z}_{\text {graph}} + \textrm{b}_c' $$

All linear transformations are initialised using the Xavier uniform initialisation scheme, and all biases are initialised to zero. Dropout is consistently applied before the final classification layer in multimodal and unimodal regimes to promote generalisation.

#### Distributed data-parallel training framework

We employ a DDP training strategy to efficiently scale model training across multiple computational units. This paradigm replicates the model on each processing unit and partitions the global training dataset so that each unit receives a unique mini-batch. This setup allows all devices to perform computation concurrently while maintaining synchronisation of the model parameters.

Let $\mathcal {D} = \{ \mathcal {D}_1, \ldots , \mathcal {D}_N \}$ denote the partitioning of the data set among *N* processing units. In each training step, device $i \in \{1, \ldots , N\}$ computes the gradient $\nabla \mathcal {L}_i(\theta )$ of the local loss function $\mathcal {L}_i$ with respect to the model parameters $\theta$, using its local data partition $\mathcal {D}_i$:

$$ \nabla \mathcal {L}_i(\theta ) = \frac{1}{|\mathcal {D}_i|} \sum _{(x_j, y_j) \in \mathcal {D}_i} \nabla _\theta \ell (f_\theta (x_j), y_j) $$

where $\ell (\cdot ,\cdot )$ is the task-specific loss function and $f_\theta$ denotes the model.

After computing the local gradients, a global synchronisation step aggregates the gradients across all devices:

$$ \nabla \mathcal {L}(\theta ) = \frac{1}{N} \sum _{i=1}^N \nabla \mathcal {L}_i(\theta ) $$

This synchronised gradient is then used to perform a consistent parameter update across all devices, ensuring that:

$$ \theta ^{(t+1)} = \theta ^{(t)} - \eta \nabla \mathcal {L}(\theta ^{(t)}), $$

where $\eta$ is the learning rate.

In distributed training, the accelerator module synchronises the model, the optimiser, and the data loaders while overseeing the allocation of computational resources and distributed sampling. This helps avoid data overlap and maintains the training workflow (Li et al., 2020). By managing gradient calculations, attention can be paid to refining the model without worrying about device-specific challenges, thereby improving computational efficiency and scalability. By using a hierarchical all-reduce protocol, the synchronisation process across devices decreases latency and bandwidth consumption, facilitating nearly linear performance scaling with the increased number of devices, thereby enabling scalable training.

## Model evaluation and results

### Baseline

To rigorously evaluate the performance of our proposed PlantGraphNet architecture, we compared it with a diverse set of baseline convolutional neural network (CNN) architectures widely used in image classification. These baselines include lightweight and deep networks, as well as encoder-decoder architectures. All models are initialised with ImageNet-pretrained weights, and their final classification layers are replaced and fine-tuned for the target number of plant species classes. The backbone layers are frozen to ensure fair comparison, and only the modified classification heads are trained.

- AlexNet (Krizhevsky et al., 2017): Convolutional neural networks (CNNs) consisting of sequential convolutional and fully connected layers. For the classification task, the original classifier heads are extended with additional non-linear transformations and regularisation techniques such as dropout.
- ResNet50 (He et al., 2016) and ResNet101 (He et al., 2015): Deep residual networks employing skip connections to facilitate gradient flow and improve convergence in deeper architectures. The final classification layers are replaced with multi-layer perceptrons (MLPs) to accommodate the target domain.
- GoogLeNet (Szegedy et al., 2015): A CNN based on Inception modules, which combine multiple convolutional filter sizes in parallel to capture multi-scale features within the same layer.
- YOLOv8 (Vaghela et al., 2025): An object detection architecture characterised by an anchor-free design, distinct detection heads, and improved backbone efficiency. It has been adapted for classification through the substitution of detection-specific elements with a global pooling and classification head.

All baseline models are implemented in PyTorch and share a unified training pipeline for consistency in optimisation and evaluation. Performance is measured using classification accuracy, precision, and the F1 score on the same plant species dataset used to evaluate PlantGraphNet.

### Settings

The training process is guided by the categorical cross-entropy loss, a widely adopted objective function for multi-class classification. Let $\textrm{y} \in \{0, 1\}^C$ denote the vector of ground truth encoded with a one-hot vector, and let $\hat{\textrm{y}} \in [0,1]^C$ denote the predicted probability vector after softmax activation, where *C* is the number of classes. The cross-entropy loss for a single sample is given by:

$$ \mathcal {L}(\textrm{y}, \hat{\textrm{y}}) = -\sum _{c=1}^C y_c \log (\hat{y}_c) $$

The model is optimised using the Adam optimiser, which computes adaptive learning rates for each parameter by maintaining estimates of the gradients’ first and second moments, thus facilitating stable and fast convergence across a wide range of deep learning models. During each epoch, model weights are updated based on backpropagated gradients, and the loss is accumulated to track convergence. The model achieving the lowest training loss is checkpointed for subsequent evaluation.

### Evaluation metrics

Model performance is evaluated using standard classification metrics, computed per class and aggregated as needed. We use the following:

- **Precision** (positive predictive value), defined for class *c* as:
  $$ \text {Precision}_c = \frac{TP_c}{TP_c + FP_c} $$
  where $TP_c$ is the number of true positives and $FP_c$ the number of false positives for class *c*.
- **Recall** (sensitivity or true positive rate), defined as:
  $$ \text {Recall}_c = \frac{TP_c}{TP_c + FN_c} $$
  where $FN_c$ is the number of false negatives for class *c*.
- **F1 score**, the harmonic mean of precision and recall:
  $$ \text {F1}_c = 2 \cdot \frac{\text {Precision}_c \cdot \text {Recall}_c}{\text {Precision}_c + \text {Recall}_c} $$
- **Accuracy**, the overall proportion of correctly classified instances, defined as:
  $$ \text {Accuracy} = \frac{1}{N} \sum _{i=1}^N \left( \text {if } \hat{y}_i = y_i \text { then } 1 \text { else } 0 \right) $$
  where *N* is the total number of samples, the summation adds 1 for each correctly predicted label $\hat{y}_i$; otherwise, it adds 0.

To qualitatively assess classification performance, we also use a confusion matrix. Each entry (*i*, *j*) in the matrix corresponds to the number of instances in class *i* predicted as class *j*. Finally, a comprehensive classification report is compiled, presenting the aforementioned metrics for each class individually, along with their weighted average counterparts. This detailed analysis helps us to understand class-specific behaviour.

### Results

Table 1 presents the performance of PlantGraphNet compared to baseline models. ResNet50 achieved the highest performance among conventional architectures, with an accuracy of 97.86%, alongside precision of 0.9788, recall of 0.9786, and an F1 score of 0.9785. The lightweight YOLOv8n model further improved results, reaching an accuracy of 98.95%. PlantGraphNet achieves an accuracy of 98.97%, precision of 0.9898, recall of 0.9897, and an F1 score of 0.9897. This performance highlights the advantages of combining graph-based relational modeling with convolutional feature extraction, enhancing both local and global reasoning for fine-grained vegetation classification. As indicated in Table 2, PlantGraphNet consistently performs well across classes, particularly in dominant categories like *grass* and *nonveg*. It retains strong precision and recall in challenging classes such as *lichens* and *rosa_rugosa*, though with slight degradation in performance.

**Table 1:** Overall result comparisons. Bold entries represent best results

Model	Accuracy	Precision	Recall	F1 score
VGG16	0.9560	0.9583	0.9560	0.9533
VGG19	0.9388	0.9419	0.9388	0.9344
ResNet50	0.9786	0.9788	0.9786	0.9785
ResNet101	0.9609	0.9631	0.9609	0.9570
MobileNetV2	0.9589	0.9608	0.9589	0.9576
GoogleNet	0.9556	0.9556	0.9556	0.9553
AlexNet	0.9498	0.9608	0.9576	0.9576
YOLOv8n	0.9895	0.9896	0.9895	0.9894
PlantGraphNet	**0.9897**	**0.9898**	**0.9897**	**0.9897**

**Table 2:** Classification report of *PlantGraphNet*

Class name	Precision	Recall	F1 score
Amm	0.9960	1	0.9980
Calluna	0.9886	0.9914	0.9900
Empetrum	1	0.9863	0.9930
Grass	1	0.9985	0.9992
Lichens	1	0.9285	0.9629
Myrica	0.9728	0.9748	0.9738
Nonveg	1	1	1
Rosa rugosa	0.9583	1	0.9787
Salix	0.9838	0.9777	0.9808
Trees	0.9556	0.9584	0.9570
Weighted Avg	0.9898	0.9897	0.9897

**Fig. 6:**
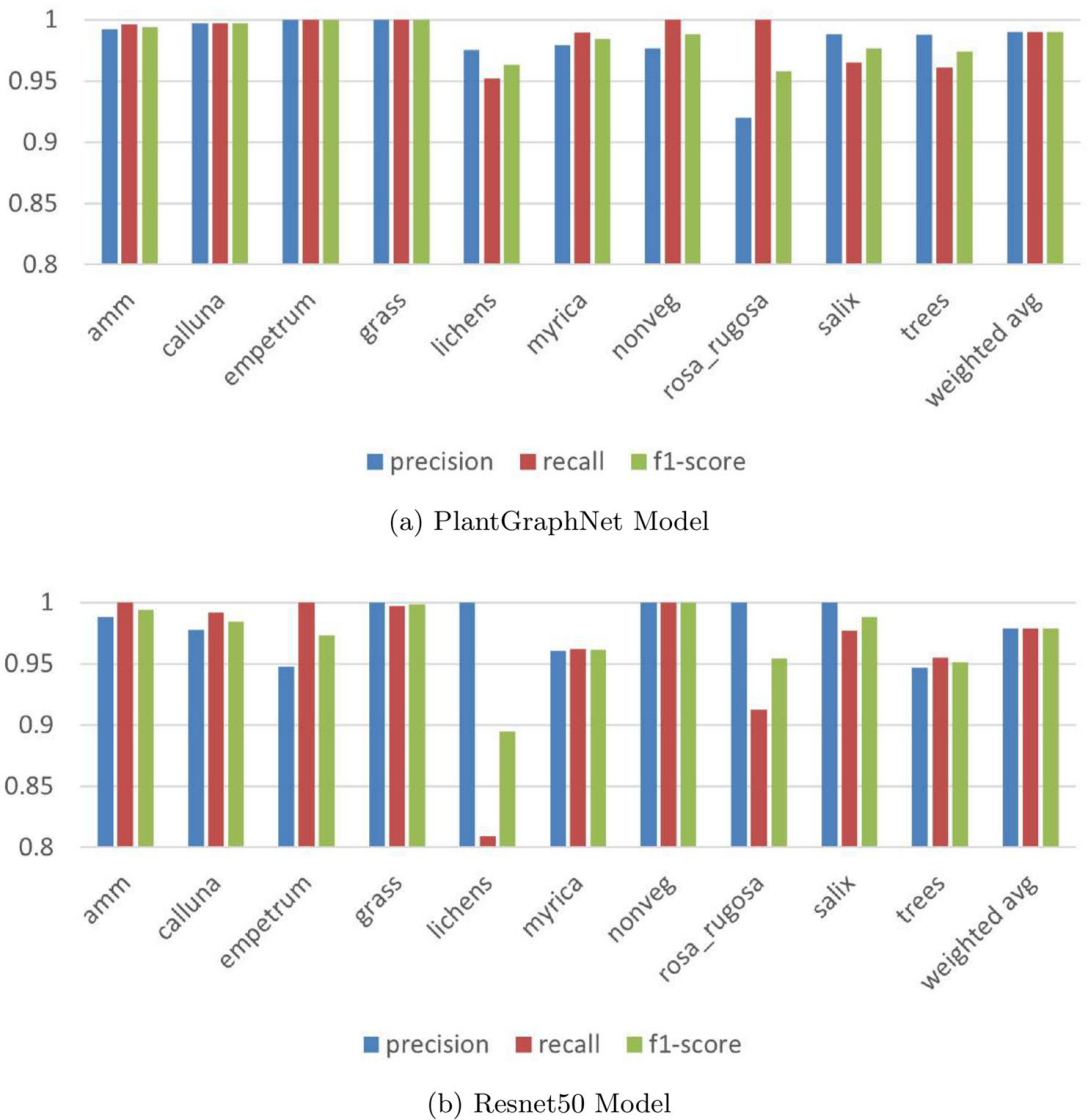
Class-wise performance between ResNet50 and PlantGraphNet

An evaluation of class-wise performance between ResNet50 and PlantGraphNet (Fig. 6) indicates that PlantGraphNet achieves more consistent performance among vegetation classes, with improved aggregate metrics including precision, recall, and F1 score. Despite both models demonstrating commendable overall performance, ResNet50 displays discernible deficiencies in specific classes, such as *lichens* and *rosa_rugosa*, where a marked decline in its recall and precision is evident. In contrast, PlantGraphNet achieves near-perfect scores in most classes, including those where ResNet50 performs poorly. Moreover, aggregate measures—such as accuracy, macro average, and weighted average—are markedly enhanced with PlantGraphNet, achieving approximately 99.5 % compared to ResNet50’s approximately 97.5 %. These findings highlight the efficacy of integrating graph-based relational information within vegetation classification tasks.

**Fig. 7:**
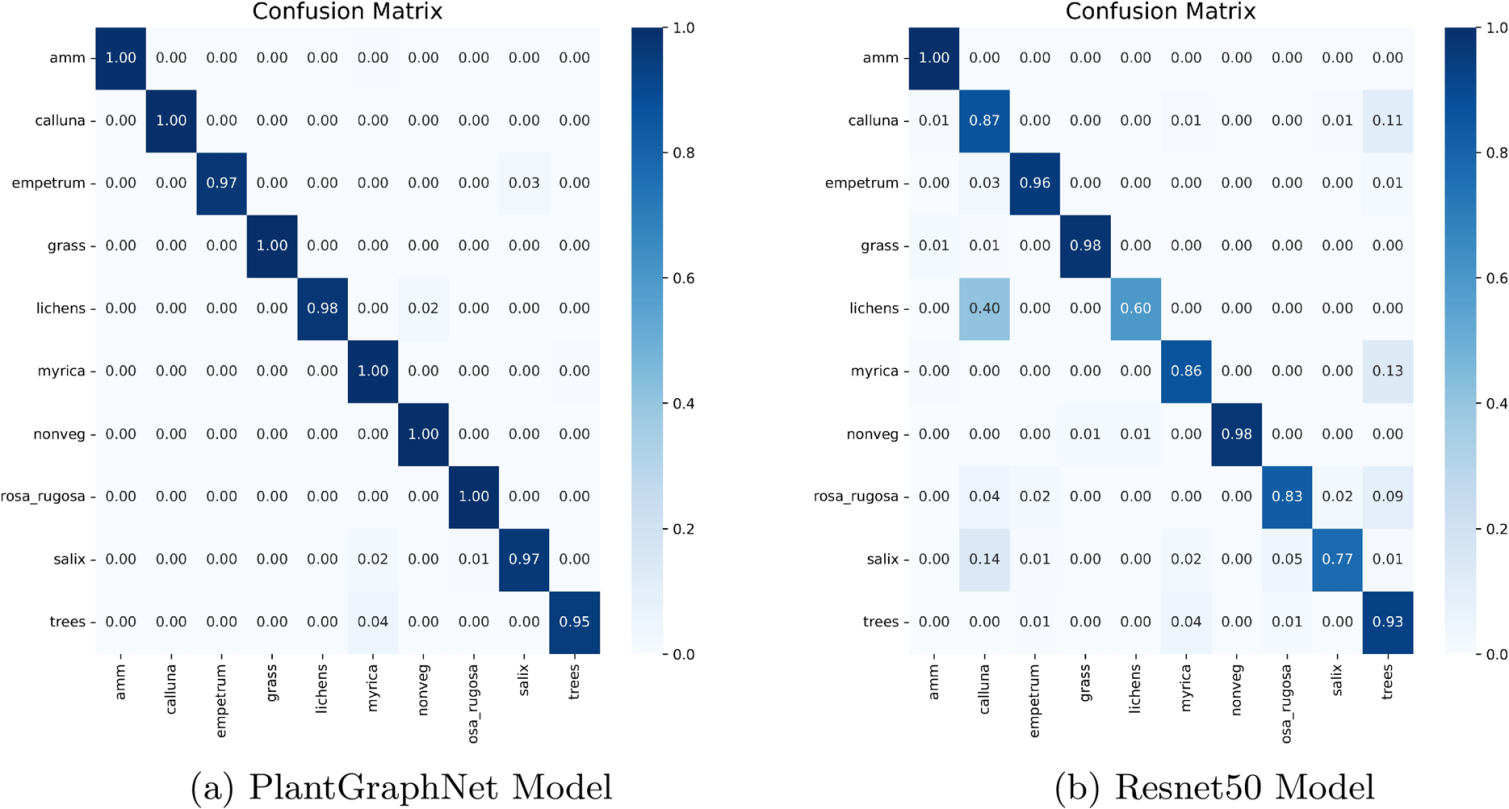
Confusion matrix of Resnet50 vs PlantGraphNet

The confusion matrix analysis supports this finding. Figure 7 shows that PlantGraphNet has superior precision with more diagonal concentration, indicating consistent correct predictions. Conversely, ResNet50 shows more off-diagonal elements, indicating greater confusion. These differences highlight PlantGraphNet’s effective use of graph-based features to distinguish similar inputs better. Overall, PlantGraphNet outperforms a conventional CNN backbone in classification. Although YOLOv8n achieves strong accuracy with lower modelling complexity, it is adapted from an object detection architecture and does not explicitly encode spatial relationships between plant structures. PlantGraphNet instead prioritises relational reasoning and interpretability, which are relevant in ecological analysis beyond raw classification accuracy.

**Fig. 8:**
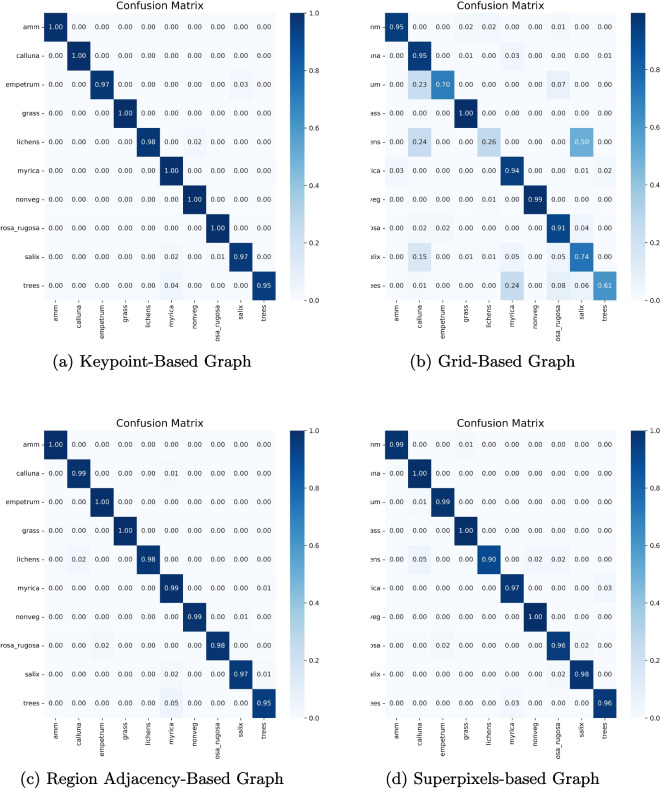
Confusion matrix of GNN-based methods using different graph construction methods

### Ablation study

#### Influence of graph construction method

We investigated the effect of graph construction strategies on model performance by comparing our proposed keypoint-based graph with three alternatives, each encoding distinct structural priors.

- Region adjacency graph (RAG): Constructs nodes from superpixel segments using SLIC, with edges formed between adjacent regions and edge weights based on mean colour dissimilarity.
- Superpixel graph: Similar to RAG, but with adjacency derived explicitly from boundary connectivity rather than a region adjacency graph, yielding a finer representation of local transitions.
- Grid graph: A dense pixel-level graph with raw RGB features and a 4-connected topology, preserving spatial locality but lacking abstraction.

The analysis of the confusion matrices, presented in Fig. 8, indicates that graph construction methods that use spatial abstraction (RAG, Superpixel Graph, and Keypoint-based graph) perform significantly better than the dense grid graph at the pixel level. This highlights the importance of structuring the input data into meaningful components beyond raw pixels for the task of plant species classification. The key point-based graph consistently performs superiorly or equally, outperforming in challenging classifications such as lichens and Rosa rugosa.

**Fig. 9:**
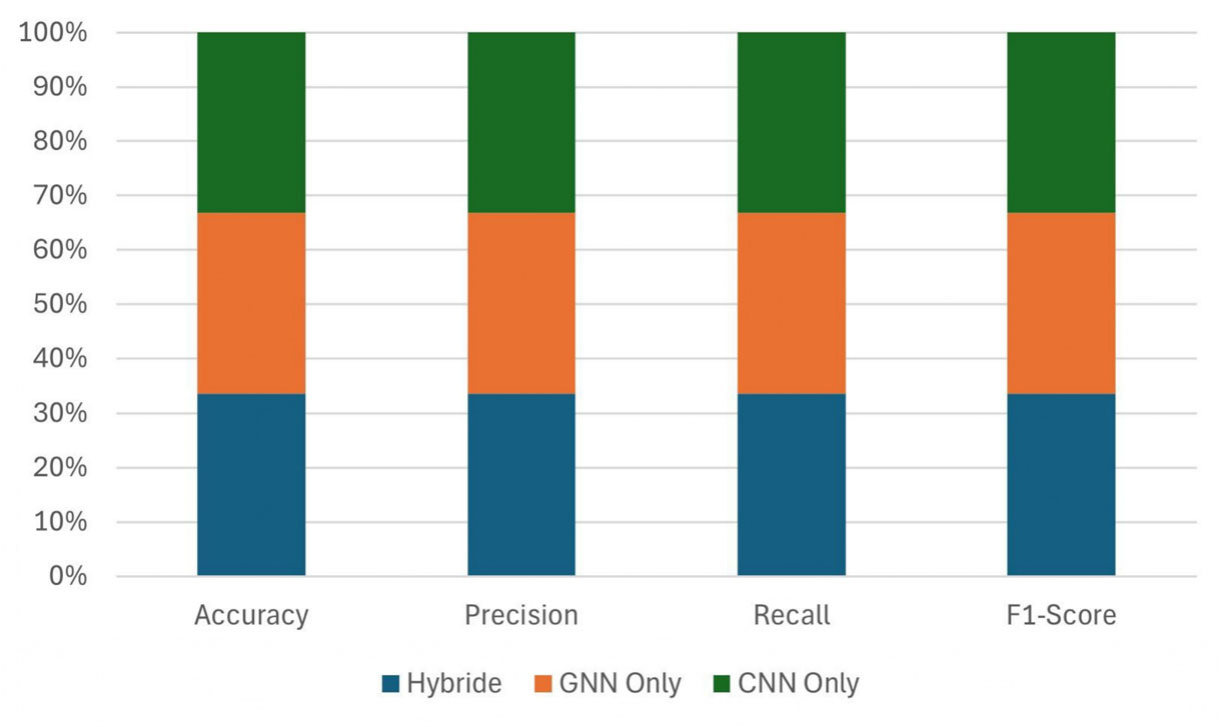
Comparison of CNN-only, GNN-only, and hybrid configurations. The CNN-only model captures local appearance cues, while the GNN-only model captures relational structure. The hybrid model fuses both, consistently outperforming the unimodal variants and improving classification accuracy, demonstrating the effectiveness of multimodal fusion for modeling plant structures

#### Influence of the hybrid model

To assess the efficacy of the proposed hybrid GNN-CNN architecture, we conducted ablation studies comparing three configurations: (i) GNN-only, utilising solely graph-structured inputs; (ii) CNN-only, leveraging only image features; and (iii) the hybrid model, which integrates both modalities through late fusion. (Fig. 9) compares CNN-only, GNN-only, and hybrid configurations. The CNN-only model captures local appearance cues, while the GNN-only model captures relational structure. The hybrid model integrates both modalities and consistently outperforms the unimodal variants, demonstrating that performance gains arise from multimodal fusion. The hybrid model combines the strengths of both GNN and CNN, thereby improving classification accuracy and demonstrating the effectiveness of the hybrid model in modelling plant structures.

**Fig. 10:**
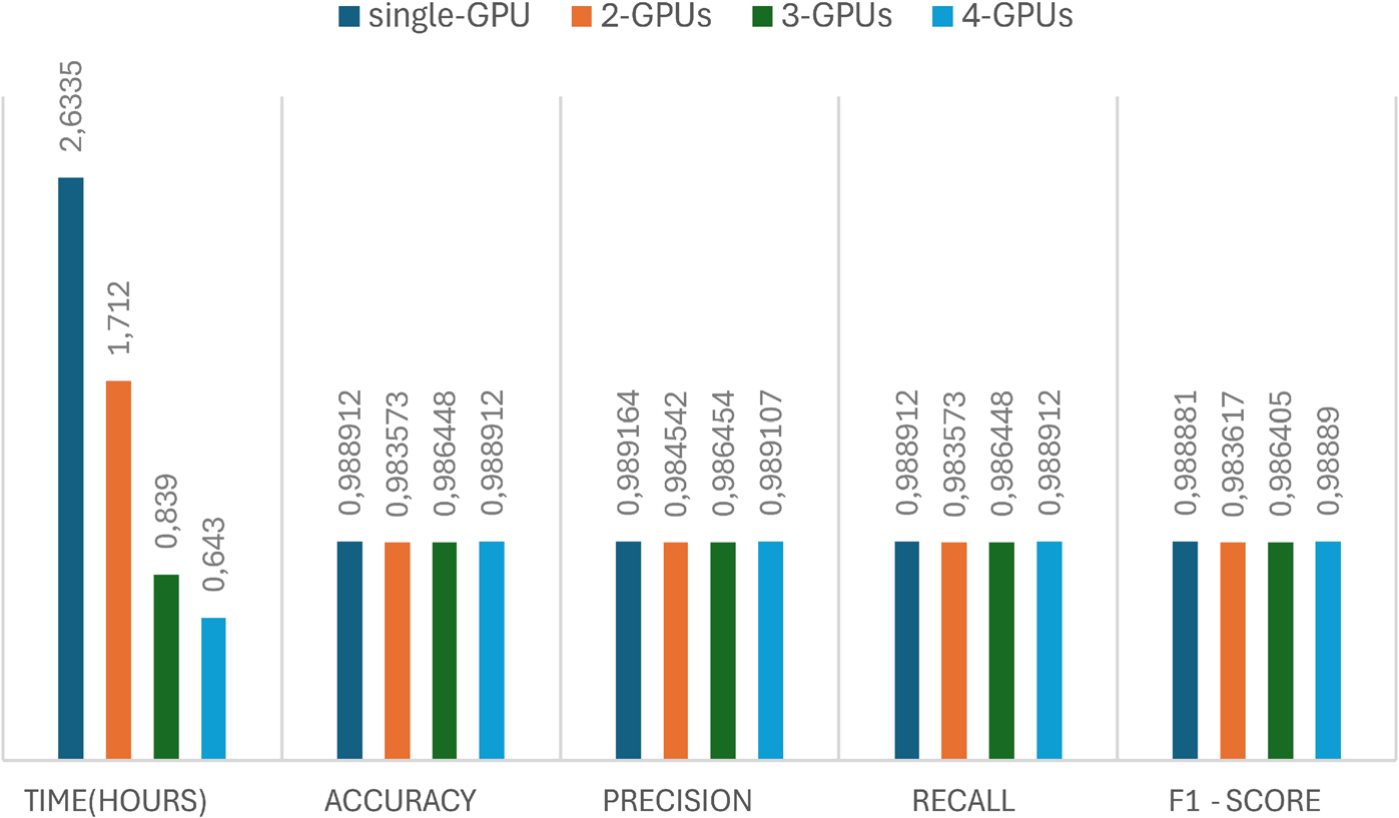
Impact of distributed data-parallel training on accuracy, recall, precision, F1 score, and training time for 1–4 GPU configurations

#### Influence of distributed data-parallel training

The effectiveness of distributed data-parallel training (DDP) for the PlantGraphNet model is evaluated using single-GPU, 2-GPU, 3-GPU, and 4-GPU configurations. The findings, depicted in Fig. 10, indicate that DDP facilitates substantial improvements in training efficiency while preserving model convergence and generalisation capabilities. As the quantity of utilised GPUs increases, the wall-clock training time diminishes almost linearly, evidencing this approach’s scalability. This acceleration is achieved while maintaining uniform convergence patterns, as indicated by the analogous metric outcomes observed across all configurations. The enhanced throughput provided by DDP permits larger effective batch sizes, consequently generating more stable gradient estimates and mitigating optimisation noise. These enhancements are particularly pertinent in contexts involving high-resolution input images and expansive graph data, where single-device operations often hinder performance.

We evaluate the impact of Distributed Data-Parallel by studying the scalability and convergence of the hybrid model with various compute nodes, focusing on training time, throughput, and model accuracy and loss for single-node and multi-node setups. Empirical evidence suggests that DDP provides a near-linear speed-up with GPU count, significantly reducing training time while ensuring stable convergence. Gradient synchronisation among replicas ensures consistent updates, and distributed sampling eliminates data redundancy, resulting in stable generalisation with minimal variance in validation metrics. By supporting larger batch sizes, DDP improves the efficiency of training data, enhances the precision of gradient estimation, and reduces optimisation noise.

## Limitations

The hybrid GNN-CNN model exhibits strong performance; however, it faces several challenges. The fixed *k*-nearest-neighbour approach may lead to suboptimal graph representations, especially when keypoint densities vary. Adaptive or learnable graph construction strategies could mitigate this limitation in future work. Despite being evaluated on heathland data, the proposed hybrid GNN–CNN framework is applicable to other morphology-driven domains such as forestry and crop monitoring. Additionally, its current application is limited to static 2D images, reducing its utility in dynamic or 3D scenarios. Improvements can be achieved by refining the graph parameters, altering the neighbour count *k*, and employing various edge weighting strategies. Enhancing the GNN/CNN framework, fine-tuning hyperparameters, and adjusting the learning rate are expected to improve generalisation.

## Conclusion

We present PlantGraphNet, a novel graph-based framework for classifying plant species that captures structural and contextual morphological cues in a modular and extensible manner. Our architecture systematically models spatial and morphological relationships using structured graphs, thus capturing topological information that CNNs typically overlook. Empirical evaluations indicate consistent and substantial improvements over standard baselines, highlighting the essential role of relational reasoning in ecologically complex classification tasks. We use distributed data-parallel training to ensure scalability, facilitating efficient optimisation across large datasets without compromising convergence or generalisation. While our evaluation focuses on single-label classification, our framework is adaptable to multilabel contexts, making it suitable for ecological settings involving species co-occurrence and interaction. PlantGraphNet thus provides a robust, scalable, and ecologically informed approach to automated biodiversity evaluation, laying the groundwork for structurally aware deep learning in environmental monitoring.

## Data Availability

The datasets generated during and/or analysed during the current study are available from the corresponding author on reasonable request.

## Footnotes

1 https://www.google.com/maps?q=57.12317,9.01179
